# Promising application of probiotic microorganisms as Pickering emulsions stabilizers

**DOI:** 10.1038/s41598-023-43087-w

**Published:** 2023-09-23

**Authors:** Maryam Nejadmansouri, Mohammad Hadi Eskandari, Gholam Hossein Yousefi, Masoud Riazi, Seyed Mohammad Hashem Hosseini

**Affiliations:** 1 Department of Food Science and Technology, School of Agriculture, Shiraz University, Shiraz, Iran; 2 Department of Pharmaceutics, School of Pharmacy, Shiraz University of Medical Sciences, Shiraz, Iran; 3 Enhanced Oil Recovery (EOR) Research Centre, IOR/EOR Research Institute, Shiraz University, Shiraz, Iran; 4 Department of Petroleum Engineering, School of Chemical and Petroleum Eng, Shiraz University, Shiraz, Iran

**Keywords:** Applied microbiology, Bacteria, Industrial microbiology

## Abstract

The purpose of this work was to study the ability of nineteen food-grade microorganisms as Pickering emulsion (PE) stabilizers. Medium-chain triacylglycerol (MCT) oil-in-water (50:50) PEs were fabricated by 10 wt% or 15 wt% of thermally-inactivated yeast, cocci, *Bacillus* spp. and lactobacilli cells. The characteristics of microorganisms related to “Pickering stabilization” including morphology, surface charge, interfacial tension, and “contact angle” were firstly studied. After that, the cells-stabilized PEs were characterized from both kinetic and thermodynamic viewpoints, microstructure and rheological properties. The interfacial tension and “contact angle” values of various microorganisms ranged from 16.33 to 38.31 mN/m, and from 15° to 106°, respectively. The mean droplet size of PEs ranged from 11.51 to 57.69 µm. Generally, the physical stability of cell-stabilized PEs followed this order: lactobacilli > *Bacillus* spp. > cocci > yeast. These variations were attributed to the morphology and cell wall composition. Increasing the microorganism concentration significantly increased the physical stability of PEs from a maximum of 12 days at 10 wt% to 35 days at 15 wt% as a result of better interface coverage. Shear-thinning and dominant elastic behaviors were observed in PEs. Physical stability was affected by the free energy of detachment. Therefore, food-grade microorganisms are suggested for stabilizing PEs.

## Introduction

Pickering emulsions (PEs) are a type of emulsions which are stabilized by the accumulation of insoluble solid particles at the interface^1^. The advantages of PEs over surfactant-stabilized emulsions include higher stability against coalescence and disproportionation, better taste characteristics, and the irreversible adsorption of solid particles to the interface^2,3^. The high physical stability of PEs against coalescence is rooted in strong anchoring energy, specific inter-particle interactions, and formation of densely packed particle layers at the interfaces^4^. The adsorption and desorption energy of particles at the interface is of the order of k_B_T (which is known as the thermal energy of Brownian motion)^5^ (Eq. 1)

1 $${\text{E}} = {\text{k}}_{{\text{B}}} {\text{T}}$$

where, E is the thermal energy (J); k_B_ is the Boltzmann constant (J/K) and T is the absolute temperature (K). A dynamic balance of adsorption and desorption from the interface is observed for the surfactant molecules, however, for solid particles, the attachment at the interface is irreversible when the free energy of detachment (Δ*G*_d_) is much greater than k_B_T^5^. The adsorption might be a reversible process whenever Δ*G*_d_ is lower than 10 × k_B_T^6^. For most PEs stabilized by particles with appropriate “contact angles” (*θ*_ow_) (i.e., not too close to 0° or 180°), Δ*G*_d_ is several thousands of thermal energy and thus the attachment is almost irreversible^5^.

Many types of food-grade and non-food-grade particles are utilized as Pickering stabilizers. Among food-grade Pickering stabilizers, biopolymer-based (e.g., starch granules, and protein aggregates) and microorganism-based particles can be utilized for the formation of PEs^6,7^. Some biopolymer-based particles need further pretreatments (e.g., controlled aggregation of protein, and complex coacervation between protein and polysaccharide) for the formation, which can make their application in PE stabilization more challenging^8^. As potential Pickering particles, microorganisms do not require any specific step for their preparation (except for culturing that can be done in bioreactors)^9^. Due to safety concerns and also providing added values to final products, food-grade probiotic microorganisms are the solid particles of choice for the preparation of Pickering food emulsions. Considering the potential probiotic and prebiotic activity of live and inactive microorganisms, their utilization in food emulsion products can provide additional advantages to the consumers^10^. Similar to other Pickering particles, the size, shape, and surface characteristics of microorganisms are of importance for effective “Pickering stabilization”. Microorganisms have different geometric shapes (e.g., rod, cocci and ellipsoid) that can influence their adsorption at the interface, and thus the stability of emulsions^11^. A wide range of particle size can be utilized in emulsion stabilization. Higher physical stability is achieved when the size of particles is smaller than that of the oil droplets, possibly due to a higher packing density and homogeneity at the interface^12^. The size of microorganism also influences the desorption from the interface. Microorganisms have a higher motility and diffusivity than surfactant molecules. The motility is supported by an enhanced Brownian motion mechanism. Wettability, roughness, surface charge, and cell wall characteristics also affect the adsorption phenomenon^13,14^. Surface roughness enhances the accessible surface area. The surface charge of microorganisms is rooted in carboxyl, amino, and phosphate functional groups, which is an important factor on the adsorption to the interface. High electronegativity might decrease the bacterial adhesion to the interface, while, heterogeneity in surface charge considerably enhances the cell adhesion^13,14^. The cell wall of Gram-positive bacteria consists of thick peptidoglycan layer. The outer surface is covered by S-layer proteins, lipoteichoic acid, and polysaccharides, which help to reduce the interfacial tension (IFT) and improve PE stability^13^. The presence of negatively-charged extracellular polysaccharides (EPS) also increases the adsorption to the interface.

In addition to the Pickering particle type, there are other factors which affect the final physical and chemical stability of PE including the quantity and saturation degree of the oil phase. The volume fraction of dispersed phase (known as φ) determines the physical stability of emulsion through affecting the apparent viscosity (*η*) ratio of dispersed phase to continuous phase^15^. The medium-chain triacylglycerol (MCT) oil is a dietary oil, which can be used in the formulation of food emulsions. It is produced from some fractions of palm kernel and coconut oils^16^. The advantages of MCT oil over long-chain triacylglycerol (LCT) oil are rapid metabolism into ketone bodies and higher absorption^17^. The disadvantage of MCT oil is having a high percentage of saturated fatty acids. However, in an emulsion system, this can improve the oxidative stability of emulsion.

There are few reports dealing with the stabilization of PEs by food-grade microorganisms. Firoozmand and Rousseau (2016) studied the stabilization of O/W PEs by three types of microorganisms including *Saccharomyces cerevisiae*, *Lactobacillus acidophilus*, and *Streptococcus thermophilus* at various φ values and Pickering microorganisms’ concentrations^11^. They reported that some emulsions remained stable for more than four months. The physical stability of PEs followed this order: *Saccharomyces cerevisiae* > *L. acidophilus* > *S. thermophilus*. Moreover, Jiang et al. reported that the surface modification of *L. acidophilus* by octenyl succinic anhydride (OSA) led to improve the stability of PEs^18^. There are also some reported results regarding the utilization of non-food-grade microorganisms (e.g., *Acinetobacter venetianus*, *Rhodococcus erythropolis*, *Pseudomonas fluorescens* and *Rhizomonas suberifaciens*) in the stabilization of PEs. The highest and the lowest emulsion stability were observed in samples stabilized by *A. venetianus* (several months) and *R*. *erythropolis* (24 h), respectively. Also, *P. fluorescens* and *R. suberifaciens* were not able to stabilize the emulsions^19^. In another study, Wongkongkatep et al. studied the ability of cell/polymer networks fabricated by electrostatic attraction between positively charged chitosan and negatively charged *Escherichia coli* on the stabilization of PEs^20^.

Taking into account the large diversity of food-grade microorganisms and their differences even at the strain level, the potential application of these particles as novel candidates for stabilizing PEs should be determined. Therefore, the main objective of this work was to study the efficacy of nineteen food-grade microorganisms varying in genus, species, and strains on the stability of MCT O/W PEs. At the first stage, various characteristics of microorganisms related to “Pickering stabilization” including surface charge, IFT, *θ*_ow_, and morphology were studied. Following, PEs were produced using MCT oil as the lipid phase (at a constant φ of 0.5), and characterized in terms of physical stability, droplet size, zeta potential, microstructure, Δ*G*_d_, and rheological properties.

## Materials and methods

### Materials

MCT oil was purchased from Nutricia Ltd. (Favona, Auckland, New Zealand). The fatty acids composition of MCT oil was caprylic (8:0, 53%), capric (10:0, 36–47%) and lower amounts of caproic (6:0) and lauric (12:0) acids. De man, Rogosa and Sharpe (MRS) broth, tryptic soy broth (TSB) and Sabouraud Dextrose Broth (SDB) were purchased from Merck Co. (Darmstadt, Germany). Chloramphenicol (purity > 99%) was purchased from Solarbio (Beijing, China). 100 μL of chloramphenicol ethanolic solution (0.1 g/1 mL EtOH) was added to 100 mL of SDB culture medium.

### Preparation of microorganisms

More information about the genus, species, strains, culture medium, incubation temperature and incubation time of nineteen microorganisms is reported in Table 1. From different glycerol stocks, each microorganism was cultured on respective culture medium (agar plate) and incubated at appropriate temperature for 24 h to acquire a pure single colony. Active pure cultures were then obtained by taking a single colony from the culture medium followed by inoculation in 5 mL of culture medium broth in a shaking incubator. The incubation conditions were as follows: 150 rpm at 37 °C for lactobacilli, *Bacillus* spp., and cocci bacteria and 200 rpm at 28 °C for yeast cells. After that, an aliquot of 1 mL from the prepared subculture was inoculated into 10 mL of respective culture media and incubated in similar conditions. Then 10 mL of preculture was transferred into 100 mL of fresh medium and incubated until the optical density at 600 nm (OD_600_) reached 2. Finally, microorganisms were harvested by centrifugal force at 10,000×*g* for 5 min at 25 °C, washed twice times with sterile saline solution, and inactivated in a water bath at 85 °C for 30 min for lactobacilli and cocci bacteria and also yeast cells and at 121 °C for 15 min for *Bacillus* spp. The samples were then rapidly cooled to room temperature and subjected to centrifugation in similar conditions. After removing the supernatant, inactivated cells were washed five times with sterile saline solution and centrifuged. At the final stage, the inactivated microorganisms were washed with phosphate buffer solution (PBS 5 mM, pH 6.8). Uniform cells (non-lyophilized wet sediments) were obtained after centrifugation and then stored at 4° C for characterization. A fraction of wet sediments was lyophilized and then kept at room temperature for the preparation of PEs.

**Table 1:** Microorganisms studied in this work.

Genus	Species	Strains	Culture medium	Incubation temperature (°C)	Incubation time (h)
*Enterococcus*	*faecium*	(BH06)	MRS	37	24
*Pediococcus*	*acidilactici*	(M76)	MRS	37	24
*Lactobacillus*	*delbrueckii*	(PTCC 1743)	MRS	37	24
*Lactiplantibacillus*	*plantarum*	(Lp 299)	MRS	37	24
*Lactiplantibacillus*	*plantarum*	(ATCC 8014)	MRS	37	24
*Lactiplantibacillus*	*plantarum*	(PTCC 1058)	MRS	37	24
*Lacticaseibacillus*	*casei*	(ATCC 393)	MRS	37	24
*Lacticaseibacillus*	*rhamnosus*	*GG* (ATCC 53103)	MRS	37	24
*Lactobacillus*	*acidophilus*	(ATCC 4356)	MRS	37	24
*Limosilactobacillus*	*reuteri*	*DSM* 20016 (ATCC 23272)	MRS	37	24
*Limosilactobacillus*	*reuteri*	*DSM* 17939	MRS	37	24
*Lactobacillus*	*gasseri*	(ATCC 33323)	MRS	37	24
*Bacillus*	*subtilis*	(DE111)	TSB	37	24
*Bacillus*	*coagulans*	(MTCC 5856)	TSB	37	24
*Bacillus*	*licheniformis*	(ATCC 14580)	TSB	37	24
*Bacillus*	*indicus*	(HU36)	TSB	37	24
*Saccharomyces*	*cerevisiae*	(PTCC 5052)	SDB	28	24
*Saccharomyces*	*boulardii*	(ATCC MYA-797)	SDB	28	24
*Saccharomyces*	*boulardii*	(ATCC 18824)	SDB	28	24

### Microorganism characterization

#### Scanning electron microscopy

Prior to morphological evaluation by scanning electron microscopy (SEM, TESCAN-Vega 3, TESCAN Co., Czech Republic), air-dried non-lyophilized wet sediments were coated by a thin layer of gold (Desk Sputter Coater DSR1, Nanostructural Coating Co., Iran). The morphology of microorganisms was evaluated at an accelerating voltage of 20 kV and a magnification of 22.5 kx^21^.

After taking the micrographs, the sphericity was measured according to Eq. (2)^22^.

2 $$\mathrm{Sphericity}={\left(\frac{Volume \; of \; solid \; sample}{Volume \; of \; circumscribed \; sphere }\right)}^{1/3}$$

The volume of rod-shaped microorganisms (lactobacilli and *Bacillus* spp.) was calculated as *πr*^*2*^*L*, and the volume of cocci- and ellipsoid-shaped microorganisms (cocci, yeast) was calculated as (*4/3)πr*^3^.

#### Zeta potential

The non-lyophilized wet sediments were dispersed in double distilled water (DDW) to reach an OD_600_ of 0.7. The zeta potential of microorganisms was determined by dynamic light scattering (DLS, SZ100, Horiba, Japan) at 25 °C^23^.

#### Interfacial tension

Dispersion of non-lyophilized wet sediments was prepared in PBS so that to reach an OD_600_ of 0.7. Static and dynamic IFT were determined in MCT oil using the pendant drop method (Drop shape analyzer 100, KRÜSS GmbH, Hamburg, Germany). To determine static IFT, a drop of aqueous phase was formed at the tip of a needle in bulk MCT oil and IFT was measured exactly at the moment that the droplet detached from the needle. To determine dynamic IFT, a droplet of the aqueous phase with a constant volume of 10 µL was formed at the tip of needle, then the injection was stopped and dynamic IFT was measured by monitoring the changes in the shape of droplet through edge detection and fitting Laplace–Young Eq. ^24^. The dynamic IFT was only measured for four microorganisms. Actually, each selected microorganism represents a group of those studied in this work (*E. faecium* (cocci), *L. delbrueckii* (lactobacillus), *B. licheniformis* (bacillus) and *S. boulardii* (yeast)).

#### Contact angle

*θ*_ow_ value of non-lyophilized wet sediments was measured by a drop shape analyzer. A flat layer of pellet was placed at the bottom of glass chamber and then carefully covered with the MCT oil. Then, water droplets (2 µL) were deposited on the pellet layer and photographed by a CCD camera. *θ*_ow_ was determined by Image J software (ver. 1.53)^20^.

#### Free energy of detachment

The minimum energy required for the detachment of Pickering particles from the O/W interface is known as Δ*G*_d_. It is calculated by Eq. (3)^1^.

3 $$\Delta G_{{\text{d}}} = \pi r^{{2}} \times \gamma_{{{\text{ow}}}} \times \left( {{1} - |{\text{cos}}\theta_{{{\text{ow}}}} |} \right)^{{2}}$$

where, *r* is the particle radius; *γ*_ow_ is the oil–water IFT; *θ*_ow_ is the three-phase “contact angle” of particles.

### Pickering emulsion preparation

The aqueous phase containing 10 wt% or 15 wt% of lyophilized microorganisms was firstly prepared in DDW. Then, the MCT oil phase (2.5 g) was added to the aqueous phase (2.5 g) and magnetically stirred (700 rpm for 2 min). Homogenization (16,000 rpm for 10 min) was carried out using a high speed homogenizer (Heidolph Silent Crusher, Schwabach, Germany) equipped with type 8F stainless steel probe.

### Emulsion characterization

#### Emulsion type

The type of PEs was determined by the visual observation of dispersing an emulsion droplet into MCT oil or water phase^25^.

#### Emulsion activity index

PEs (1 mL) were diluted 100 times by DDW just after preparation. Then, 1 mL of diluted emulsion was mixed with 15 mL of 0.1% SDS solution. SDS was used to prevent the flocculation of emulsion droplets. Finally, the absorbance was measured at 500 nm (spec T92^+^, Pg instrument, United Kingdom). The emulsion activity index (EAI) was determined as follows^26^:

4 $${\text{EAI }}\left( {{\text{m}}^{{2}} \;{\text{g}}^{{ - {1}}} } \right) = ({2}T \times A \times {\text{dilution}}\;{\text{factor}})/\left( {c \times \varphi \times L \times {1}0000} \right)$$

where, *T* (turbidity of PE) is 2.303; *A is the* absorbance; dilution factor is 1600; *c* is the weight of microorganism per unit volume (g/mL); *φ* is the volume fraction of MCT oil (0.5); and *L* is the width of the optical path (0.01 m).

#### Droplet size

The volume-weighted average droplet size (*D*_43_) and droplet size distribution (span) of PEs, which remained stable after 4 days, were measured by laser diffraction (Mastersizer 2000, Malvern, UK)^25^. The refractive indices of MCT oil and water were 1.45 and 1.33, respectively.

#### Zeta potential

The zeta potential of diluted PEs (1:50), which remained stable after 1 day, was determined as the method described in section "Zeta potential".

#### Rheological properties

Different rheological properties of PEs, which remained stable after 1 day, including steady shear, amplitude sweep and frequency sweep tests were determined at 25 °C using a rheometer (Anton Paar MCR 302, Graz, Austria) equipped with a cone and plate geometry (diameter: 25 mm, cone angle: 1°, gap: 0.052 mm).

Steady shear test was carried out at shear rate range of 0.1–100 s^−1^. The results were analyzed using various models including Power Law, Herschel–Bulkley, Bingham, and Casson (Eqs. 5–8, respectively)^27^. Moreover, the *η* of different PEs was also reported at the shear rate of 57.6 s^−1^.

5 $$\tau = k\gamma^{{\text{n}}}$$

6 $$\tau = \tau_{0} + k\gamma^{{\text{n}}}$$

7 $$\tau = \tau_{0} + \mu \gamma$$

8 $$\tau^{0.5} = \tau_{0}^{0.5} + k\gamma^{0.5}$$

where, γ is the shear rate (s^−1^); τ is the shear stress (Pa); τ_0_ is the yield stress (Pa); *k* is the consistency coefficient (in Pa s^n^ for Herschel-Bulkley and Power Law and Pa s^0.5^ for Casson); *n* is the flow behavior index (dimensionless); and *μ* is the Bingham viscosity (Pa s).

Prior to performing frequency sweep test, the linear viscoelastic region (LVE) was determined using an amplitude (strain) sweep test at an angular frequency of 10 rad/s and strain range of 0.01–100%. The frequency sweep test was then performed at strain of 0.1% and angular frequency range of 0.1–100 rad/s.

#### Storage and freeze–thaw stability

PEs were kept at 25 °C and the storage stability (physical stability index) was evaluated over time using Eq. (9).

9 $$\mathrm{\% Physical \; stability \; index}=\left(\frac{HU}{HT}\right)\times 100$$

where, *HT* and *HU* are the total height of emulsion and upper layer, respectively.

Three emulsion samples (each from one group of microorganisms) were subjected to freeze–thaw cycling. The fresh samples were frozen at  − 22 °C for 24 h and then thawed during 24 h at + 25 °C. After thawing, samples were centrifuged at 3000×*g* for 10 min and the released liquid was separated and the retentate was subjected to another freezing cycle. The freeze–thaw cycling was studied under four cycles and evaluated as the percentage of released liquid from the initial sample^28^.

#### Emulsion microstructure

The morphology of PEs was visualized using an Olympus CH2 optical microscope (Japan). The samples were photographed using a digital camera (Optikam PRO 5, OPTIKA Co., Italy) at 40 × magnification^29^.

### Statistical Analysis

All tests were done at least in triplicate. The results were reported as mean values and standard deviations. Analysis of variance (ANOVA) was performed utilizing the SPSS software (ver. 22, IBM, New York). Duncan’s multiple ranges tests among the means were carried out at a significance level of 0.05. Also, analyzing the significant differences between Pickering cells concentrations was done based on paired-sample *t*-test.

## Results and discussion

### Characterization of microorganisms

#### Shape

**Figure 1:**
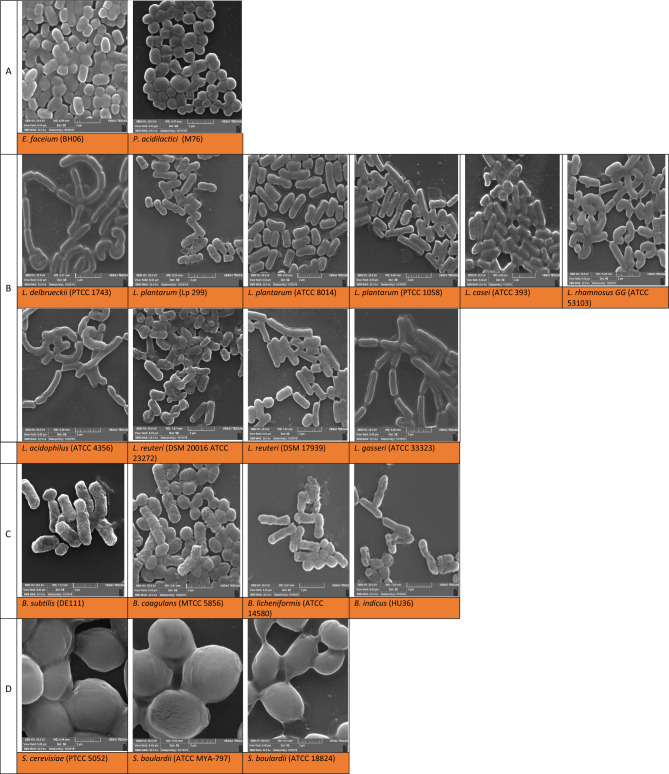
SEM micrographs of microorganisms (Scale 2 μm, Mag. 22.5 K). (**A**) Cocci cells, (**B**) Lactobacilli cells, (**C**) Spore-forming Bacilli cells, and (**D**) Yeast cells.

The shape of particles (e.g., microorganism cells) can affect the final stability of PEs. Figure 1 illustrates the morphology of different microorganisms applied in this study. The yeast cells were oval-shaped (ellipsoid) and significantly larger than the bacterial cells. While, the bacterial cells were spherical (coccus) or rod-shaped (*Bacillus* spp. and lactobacillus). The particle size of microorganisms is reported in Table S1.

Particles’ sphericity is a morphological property which affects the flow properties, heat/mass transfer^22^, as well as the wettability at the O/W interface. As shown in Table 2, the sphericity values of yeast and cocci cells were ≈ 1, while, those of lactobacilli and *Bacillus* spp. cells were in the range of 0.6–0.8. *Bacillus* spp. revealed some spherical structures along with the dominant rod-shaped structure, which might be attributed to the spore formation as a result of heat inactivation.

**Table 2:** Characteristics of microorganisms.

Names of microorganisms/groups	Zeta potential (mV)	Sphericity factor	Static IFT (mN/m)	θ (°)	Δ*G*_d_ (× 10^−15^ J)
Cocci group (cocci-shaped)					
*E. faecium* (BH06)	− 21.07 ± 1.53^i^	0.97 ± 0.09^b^	17.33 ± 1.44^p^	78.68 ± 0.31^e^	4.32 ± 0.06^g^
*P. acidilactici* (M76)	− 18.80 ± 0.56^l^	0.96 ± 0.22^b^	22.51 ± 0.94^i^	28.54 ± 1.99^m^	0.19 ± 0.02^p^
Lactobacilli group (rod-shaped)					
*L. delbrueckii* (PTCC 1743)	− 14.43 ± 1.40^p^	0.86 ± 0.25^e^	20.96 ± 1.23^k^	106.12 ± 1.43^a^	14.71 ± 0.07^e^
*L. plantarum* (Lp 299)	− 26.20 ± 1.83^a^	0.83 ± 0.24f	16.86 ± 0.01^q^	42.00 ± 1.17^k^	1.06 ± 0.00^m^
*L. plantarum* (ATCC 8014)	− 24.70 ± 2.55^e^	0.71 ± 0.07^h^	22.12 ± 1.63^j^	17.09 ± 1.29^r^	0.06 ± 0.10^q^
*L. plantarum* (PTCC 1058)	− 25.93 ± 2.05^b^	0.57 ± 0.08^k^	28.93 ± 0.17^g^	42.30 ± 1.17^j^	3.66 ± 0.13^h^
*L. casei* (ATCC 393)	− 25.13 ± 0.45^d^	0.70 ± 0.08^h^	35.09 ± 1.49^f^	24.43 ± 2.78^o^	0.32 ± 0.15^o^
*L. rhamnosus GG* (ATCC 53103)	− 17.57 ± 1.75^m^	0.77 ± 0.28^g^	19.90 ± 0.11^n^	75.16 ± 2.45^f^	14.76 ± 0.18^e^
*L. acidophilus* (ATCC 4356)	− 20.97 ± 1.00^j^	0.89 ± 0.30^d^	17.23 ± 1.58^p^	74.40 ± 1.03^g^	43.25 ± 0.50^b^
*L. reuteri* (DSM 20016, ATCC 23272)	− 21.97 ± 1.67^g^	0.65 ± 0.24^i^	18.69 ± 1.66^o^	81.53 ± 1.13^d^	16.57 ± 0.10^d^
*L. reuteri* (DSM 17939)	− 19.23 ± 0.75^k^	0.87 ± 0.25^e^	16.33 ± 0.30^r^	55.86 ± 1.15^h^	3.65 ± 0.02^h^
*L. gasseri* (ATCC 33323)	− 14.00 ± 1.45^q^	0.61 ± 0.01^j^	20.54 ± 0.13^l^	103.25 ± 1.75^b^	26.35 ± 0.10^c^
Spore-forming Bacilli group (Rod-shaped)					
*B. subtilis* (DE111)	− 21.93 ± 0.83^h^	0.78 ± 0.18^g^	38.73 ± 1.50^b^	24.76 ± 1.39^n^	1.21 ± 0.26^l^
*B. coagulans* (MTCC 5856)	− 22.83 ± 1.78^f^	0.84 ± 0.18^f^	25.77 ± 1.63^h^	87.50 ± 1.48^c^	55.06 ± 0.00^a^
*B. licheniformis* (ATCC 14580)	− 17.35 ± 1.06^n^	0.62 ± 0.01^j^	20.39 ± 1.90^m^	50.10 ± 0.15^i^	5.54 ± 0.20^f^
*B. indicus* (HU36)	− 25.43 ± 0.15^c^	0.55 ± 0.28^l^	40.08 ± 1.14^a^	31.09 ± 1.68^l^	2.49 ± 0.08^i^
Yeast group (ellipsoid-shaped)					
*S. cerevisiae* (PTCC 5052)	− 9.00 ± 1.34^s^	1.00 ± 0.12^a^	37.58 ± 1.76^e^	21.58 ± 1.35^p^	2.04 ± 0.14^k^
*S. boulardii* (ATCC MYA-797)	− 17.30 ± 1.47^o^	1.01 ± 0.12^a^	38.01 ± 1.60^d^	15.33 ± 1.43^s^	0.38 ± 0.00^n^
*S. boulardii* (ATCC 18824)	− 11.40 ± 0.28^r^	0.92 ± 0.06^c^	38.31 ± 1.20^c^	20.84 ± 1.13^q^	2.22 ± 0.21^j^

In each column different lowercase letters indicate significant differences (*p* < 0.05).

#### Zeta potential

The mobility of particles in an electric field (or electrophoretic mobility) is the consequence of three different forces including electric force, drag force, and retardation force (also known as relaxation effect)^30^. The zeta potential (determined from the electrophoretic mobility) of Pickering particles can influence the hydrophilic-lipophilic balance and thus their wettability at the interface. Moreover, higher zeta potential values result in a higher electrostatic repulsion among the oil droplets and thus a better physical stability. All microorganisms showed negative values of zeta potential with a considerable difference (p < 0.05) among different groups, species and even strains. As reported in Table 2, the zeta potential values ranged from − 9.00 ± 1.34 (*S. cerevisiae* (PTCC 5052)) to − 26.20 ± 1.83 mV (*L. plantarum* (Lp 299)). These differences were mainly attributed to the cell wall composition of microorganisms. The electric charge is derived from the ionization of functional (e.g., carboxyl, phosphate, and amino) groups under the effects of pH, ionic strength and growth media composition^31,32^. These functional groups are present along the backbone of peptidoglycan and various polyelectrolytes macromolecules such as teichuronic acid, lipoteichoic acid, lipopolysaccharides, lipoproteins, enzymes and mycolic acids^33^. Due to a greater concentration of negatively charged groups than positively charged ones, the net charge of microorganisms is negative. Several researchers reported negative zeta potential for *Lactobacillus delbrueckii ssp. Bulgaricus*^31^, *Lactobacillus rhamnosus GG*^34^, *Lactobacillus acidophilus*^35^, and *Lactobacillus johnsonii*^36^. In addition to the effect of cell wall composition on the zeta potential of microorganisms, this property is influenced by the shape characteristics (through affecting the relaxation effect)^33^, as well as the growth stage of microorganisms^37^. Particle shape might affect the zeta-potential values through affecting the specific surface area as well as the drag force during measurement^38^.

#### Interfacial tension

**Figure 2:**
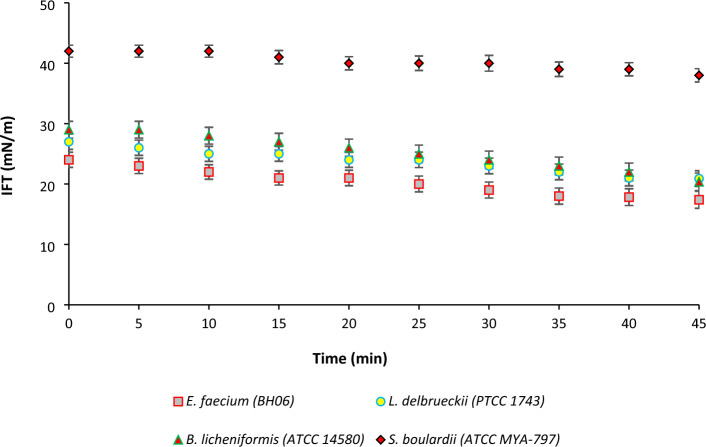
Dynamic IFT of four selected microorganisms from four groups of microorganisms (Cocci, Lactobacilli, Spore-forming Bacilli and yeast).

IFT reflects the tendency of particles for adsorption to the O/W interface^39^. A higher ability to reduce the IFT results in a greater interfacial adsorption^40^*.* As reported in Table 2, *B. indicus* (HU36) and *L. reuteri* (DSM 17939) led to the highest and the lowest static IFT values, which amounted to 40.08 and 16.33 mN/m, respectively. Microorganisms can decrease the static IFT by formation of extracellular and cell-bound biosurfactants^41,42^. The interfacial adsorption is also influenced by the surface chemistry and hydrophobicity of microorganisms^43^. The adsorption kinetics of microorganisms at the interface also depend on size and shape^44^. Smaller particles usually have faster adsorption at the interface. According to Binks et al., the rod-shaped particles generally reduce the IFT more than spherical-shaped particles due to more appropriate planar orientation and better interfacial coverage^1^. However, it seems that this conclusion cannot be readily extended to microorganisms with different morphologies. As can be seen from Table 2, the static IFT results of most cocci and lactobacilli cells were in a similar range. This means that the size and shape are not the only determinants of IFT reduction by microorganisms and other factors (such as surface smoothness and surface chemistry) might also have a contribution. For example, surface smoothness might lead to better wetting behavior as well as better spreading at the interface^1^. Despite the lower ability of some microorganisms (e.g., *B. coagulans* (MTCC 5856), *L. delbrueckii* (PTCC 1743) and *L. gasseri* (ATCC 33323)) as compared to the ability of *L. reuteri* (DSM 17939), *L. plantarum* (Lp 299), *L. acidophilus* (ATCC 4356), and *B. licheniformis* (ATCC 14580) to reduce static IFT, the respective PEs were almost stable during storage. Therefore, it can be concluded that, the IFT reduction is not the only factor in predicting “Pickering stabilization”, and other factors such as Δ*G*_d_ and rheological properties can influence the final stability (discussed later). Some researchers reported the formation of stabilized PEs without any IFT reduction^43,45^. In addition to static IFT, the dynamic IFT was also measured for four microorganisms (including *E. faecium* (BH06)*, L. delbrueckii* (PTCC 1743), *B. licheniformis* (ATCC 14580), and *S. boulardii* (ATCC MYA-797)). As shown in Fig. 2, a gradual dynamic IFT reduction was observed by *E. faecium* (BH06) from 24 to 17 mN/m*, L. delbrueckii* (PTCC 1743) from 27 to 21 mN/m and *B. licheniformis* (ATCC 14580) from 29 to 20 mN/m. However, the results showed that in the presence of *S. boulardii* (ATCC MYA-797), the droplet shape did not significantly change during the time and the dynamic IFT slightly decreased from 42 to 38 mN/m. As also verified by static IFT measurement (Table 2), the lower ability of yeasts to reduce IFT might be attributed to the larger size, ellipsoid shape and cell wall composition (polysaccharide and mannoprotein). In contrast, a gradual change (as stretching) in the shape of droplets was observed in the presence of lactobacilli, bacilli and cocci cells, confirming IFT reduction over time. The ability of bacteria to reduce IFT is arising from different surface proteins with different hydrophobicity^43^. The hydrophobicity of these three bacteria followed this order: *L. delbrueckii* (PTCC 1743) > *E. faecium* (BH06) > *B. licheniformis* (ATCC 14580). Hydrophobicity is rooted in the presence of proteins and lipoteichoic acids at the cell surface and hydrophilicity is due to the presence of polysaccharides^46^. A balance in the hydrophilic/hydrophobic properties at the cell wall determines the ability of microorganism to reduce IFT. The higher ability of rod-shaped bacteria than cocci bacteria to reduce IFT might also be related to the better interface coverage.

#### Contact angle

As a thermodynamic property, the *θ*_ow_ determines the wettability of colloidal particles by either aqueous phase or organic phase at the interface. It ranges from 0° to 180°. Lower values indicate a higher hydrophilicity and vice versa. Higher wetting by the aqueous phase (*θ*_ow_ < 90°) favors the formation of O/W PE, whereas, preferential wetting by the oil phase (*θ*_ow_ > 90°) generally results in the formation of W/O PE^47,48^. Very high hydrophilicity or hydrophobicity result in the formation of extremely unstable emulsions^2^. There are diverse parameters that affect the *θ*_ow_ value such as the size of microorganism, layer thickness, moisture content, and the assay time^49^. Significant differences (*p* < 0.05) were observed among the *θ*_ow_ values of different microorganisms (Table 2). The values ranged from 15° to 106° for *S. boulardii* (ATCC MYA-797) and *L. delbrueckii* (PTCC 1743), respectively. Some microorganisms including *E. faecium* (BH06)*, L. acidophilus* (ATCC 4356)*, L. rhamnosus GG* (ATCC 53103)*, L. reuteri* (DSM 20016, ATCC 23272)*,* and* B. coagulans* (MTCC 5856) had *θ*_ow_ values around 90° and revealed very high emulsion stability (section "Droplet size and droplet charge"). According to Chevalier and Bolzinger (2013), a great emulsion stability is observed when the *θ*_ow_ is around 90°, mainly due to the strong adsorption of particles to the interface as well as the balance between the wettability by both phases^7^. In this study, the yeast cells showed the lowest *θ*_ow_ values (around 20°), which indicated the high hydrophilicity and inability to form stable PEs. Among different groups of microorganisms, the largest variation in *θ*_ow_ values (17°–106°) were observed in lactobacilli group. These interspecies variations might be attributed to the differences in cell wall composition such as in hydrocarbon-like compounds and polysaccharide/protein ratio^50^. In this study, two species of lactobacilli microorganisms (*L. delbrueckii* (PTCC 1743) and *L. gasseri* (ATCC 33323)) had *θ*_ow_ values more than 100°, however, the respective PEs were of O/W emulsion type. Van der Mei, Bos, and Busscher. (1998) similarly reported a high *θ*_ow_ value (≈102°) for *L. acidophilus RC14*^51^*.*

### Pickering emulsion characterization

#### Droplet size and droplet charge

*D*_4,3_ of oil droplets is reported in Table 3. In this work, PE samples which showed a physical stability index of 100% after 4 days were only analyzed. A decrease in *D*_4,3_ was observed by increasing the concentration of Pickering microorganisms, which could be explained by the fact that more microorganisms were available to effectively cover the O/W interfaces^52^. Smaller oil droplets have a higher specific surface area than the larger ones and thus need more amounts of Pickering particles for effective stabilization^53^. *D*_4,3_ of PEs stabilized by cocci, lactobacilli and *Bacillus* spp. groups were in the range of 19–57, 14–57, and 11–13 µm, respectively. At 10 wt% and 15 wt% concentration, the least and the highest values were observed in the PEs stabilized by *B. licheniformis* (ATCC 14580), and *L. delbrueckii* (PTCC 1743) as well as *B. licheniformis* (ATCC 14580), and *P. acidilactici* (M76), respectively.

**Table 3:** Characteristics of Pickering emulsions stabilized by 10% (left columns) and 15% (right columns) of microorganism cells.

Names of microorganisms/group	MC (%)	Zeta potential (mV)	D_4,3_ (µm)	Span	EAI (m^2^/g)	*η* (mPa.s) at 57.6 s^−1^	MC (%)	Zeta potential (mV)	D_4,3_ (µm)	Span	EAI (m^2^/g)	*η* (mPa.s) at 57.6 s^−1^
Cocci group												
*E. faecium* (BH06)	10%	− 40.20 ± 1.17^l^*	ND	ND	327.02 ± 0.33^k^	40.7^j^	15%	− 31.03 ± 2.45^L^	19.20 ± 0.41^H^	1.52^C^	621.81 ± 0.13^D^*	214.6^H^*
*P. acidilactici* (M76)	10%	ND	ND	ND	307.58 ± 0.31^m^	ND	15%	− 57.43 ± 1.13^G^	57.69 ± 0.40^A^	1.56^B^	522.68 ± 0.36^K^*	63.9^M^
Lactobacilli group												
*L. delbrueckii* (PTCC 1743)	10%	− 35.53 ± 2.32^o^*	57.63 ± 0.35^a^*	1.42^b^	360.18 ± 0.16^h^	190.1^c^	15%	− 17.90 ± 1.55^P^	32.39 ± 0.43^B^	1.55^B^*	732.35 ± 0.26^A^*	308.3^C^*
*L. plantarum* (Lp 299)	10%	− 76.63 ± 2.15^a^*	ND	ND	298.46 ± 0.15^n^	39.1^k^	15%	− 63.03 ± 1.36^B^	20.38 ± 0.34^F^	1.56^B^	425.59 ± 0.15^M^*	189.2^I^*
*L. plantarum* (ATCC 8014)	10%	− 70.93 ± 1.13^e^*	ND	ND	277.18 ± 0.42^o^	23.8^o^	15%	− 60.10 ± 1.26^D^	ND	ND	357.37 ± 0.02^P^*	45.9^N^*
*L. plantarum* (PTCC 1058)	10%	− 64.30 ± 1.55^g^*	ND	ND	487.31 ± 0.19^b^	45.7^i^*	15%	− 45.60 ± 1.01^J^	ND	ND	607.99 ± 0.13^E^*	43.6^O^
*L. casei* (ATCC 393)	10%	− 74.50 ± 2.83^c^*	ND	ND	522.32 ± 0.10^a^	34.3^l^	15%	− 60.40 ± 1.68^C^	ND	ND	681.22 ± 0.02^B^*	158.5^J^*
*L. rhamnosus GG* (ATCC 53103)	10%	− 45.00 ± 1.91^j^*	ND	ND	309.52 ± 0.40^l^	151.0^f^	15%	− 25.37 ± 1.05^M^	30.22 ± 0.43^D^	1.48^D^	535.26 ± 0.02^J^*	704.3^B^*
*L. acidophilus* (ATCC 4356)	10%	− 36.00 ± 2.83^n^*	54.72 ± 0.39^b^*	1.44^b^	425.59 ± 0.21^e^	70.5^g^	15%	− 24.07 ± 1.18^O^	24.06 ± 0.42^E^	1.56^B^*	486.39 ± 0.11^L^*	239.5^G^*
*L. reuteri* (DSM 20016, ATCC 23272)	10%	− 44.20 ± 0.80^k^	23.61 ± 0.34^c^*	1.06^c^	432.55 ± 0.80^c^	340.3^a^*	15%	− 58.07 ± 1.08^F^*	14.93 ± 0.41^I^	1.51^C^*	671.70 ± 0.02^C^*	250.7^E^
*L. reuteri* (DSM 17939)	10%	− 45.33 ± 1.87^i^*	ND	ND	327.02 ± 0.07^k^	192.7^b^	15%	− 32.27 ± 1.61^K^	31.74 ± 0.50^C^	1.75^A^	599.88 ± 0.01^F^*	1094.9^A^*
*L. gasseri* (ATCC 33323)	10%	− 36.83 ± 1.56^m^	ND	ND	298.46 ± 0.66^n^	59.0^h^	15%	− 56.07 ± 1.40^H^*	19.61 ± 0.39^G^	1.39^E^	538.90 ± 0.06^I^*	151.3^K^*
Spore-forming Bacilli group												
*B. subtilis* (DE111)	10%	− 75.43 ± 2.74^b^	ND	ND	371.24 ± 0.19^f^*	33.2^n^	15%	ND	ND	ND	368.64 ± 0.07^O^	ND
*B. coagulans* (MTCC 5856)	10%	− 67.17 ± 2.32^f^*	11.65 ± 0.41^d^	1.45^b^	328.86 ± 0.34^j^	184.3^d^	15%	− 58.33 ± 1.20^E^	ND	ND	566.89 ± 0.00^H^*	241.6^F^*
*B. licheniformis* (ATCC 14580)	10%	− 53.70 ± 2.13^h^*	11.51 ± 0.51^d^	1.71^a^*	426.12 ± 0.25^d^	174.4^e^	15%	− 49.73 ± 1.20^I^	13.06 ± 1.03^J^*	1.33^F^	596.46 ± 0.05^G^*	262.0^D^*
*B. indicus* (HU36)	10%	− 71.63 ± 1.36^d^*	ND	ND	365.71 ± 0.42^g^	33.8^m^	15%	− 67.43 ± 1.14^A^	ND	ND	522.79 ± 0.04^K^*	90.9^L^*
Yeast group												
*S. cerevisiae* (PTCC 5052)	10%	ND	ND	ND	225.69 ± 0.05^q^	ND	15%	ND	ND	ND	317.81 ± 0.05^Q^*	ND
*S. boulardii* (ATCC MYA-797)	10%	ND	ND	ND	349.59 ± 0.03^i^	ND	15%	− 24.43 ± 1.11^N^	ND	ND	388.79 ± 0.06^N^*	43.3^P^
*S. boulardii* (ATCC 18824)	10%	ND	ND	ND	261.01 ± 0.13^p^*	ND	15%	ND	ND	ND	236.49 ± 0.04^R^	ND

For laser diffraction, Pickering emulsions which showed physical stability index of 100% after 4 days were only analyzed. For apparent viscosity and zeta potential analyses, Pickering emulsions which remained stable after 1 day were only analyzed.

*ND* not determined; *MC* microorganism concentration (%); Different lowercase and uppercase letters indicate significant differences (*p* < 0.05) among different microorganisms at the same concentration of 10 wt% and 15 wt%, respectively. For a same microorganism, the asterisk (*) shows significant differences (*p* < 0.05) between different concentrations (10 wt% and 15 wt%).

The Span value (Table 3) of all emulsions was > 1, indicating a bimodal size distribution (Fig. S1). The larger population (lying in the range of 10–100 µm) indicates the oil droplets. The smaller population (centered at ≈ 1.5 μm) likely indicated the presence of free (un-adsorbed) microorganisms in the continuous phase.

The zeta potential of oil droplets stabilized by different microorganisms ranged from − 17 to – 76 mV (Table 3). Ly et al. reported the value of − 35 mV for the emulsions stabilized by *Lactococcus lactis*^16^. For same microorganisms, the absolute value of zeta potential generally decreased by increasing the cell concentration from 10 wt% to 15 wt%. This reduction was attributed to the higher viscosity of PEs at higher cell concentration (Table 3), that could influence the electrophoretic mobility of oil droplets in the electric field. The zeta potential of microorganism- stabilized oil droplets (Table 3) was larger than the zeta potential measured in the dispersion of microorganisms (Table 2). This increase was ascribed to the packing of Pickering cells at the interface. Generally, the dispersed droplets with zeta-potential values more negative than – 30 mV and more positive than + 30 mV are colloidally stable as a consequence of sufficient electrostatic repulsion among them^54^. Some microorganism-stabilized oil droplets (e.g., by *L. delbrueckii* (PTCC 1743) and *L. acidophilus* (ATCC 4356)) showed appropriate physical stability (7 days) with zeta potential values of about − 35 mV. However, some others (e.g., by *B. subtilis* (DE111), and *L. plantarum* (ATCC 8014)) revealed lower stability (1 day) despite more negative zeta potential values (≈ − 70 mV). This observation leads to a conclusion that the zeta potential is not the only factor determining the emulsion stability during storage and other parameters (e.g., Pickering particle size, IFT reduction, and *θ*_ow_ which are collectively discussed in terms of Δ*G*_d_ as well as rheological properties) also affect the emulsion stability.

#### Emulsion formation and physical stability

**Figure 3:**
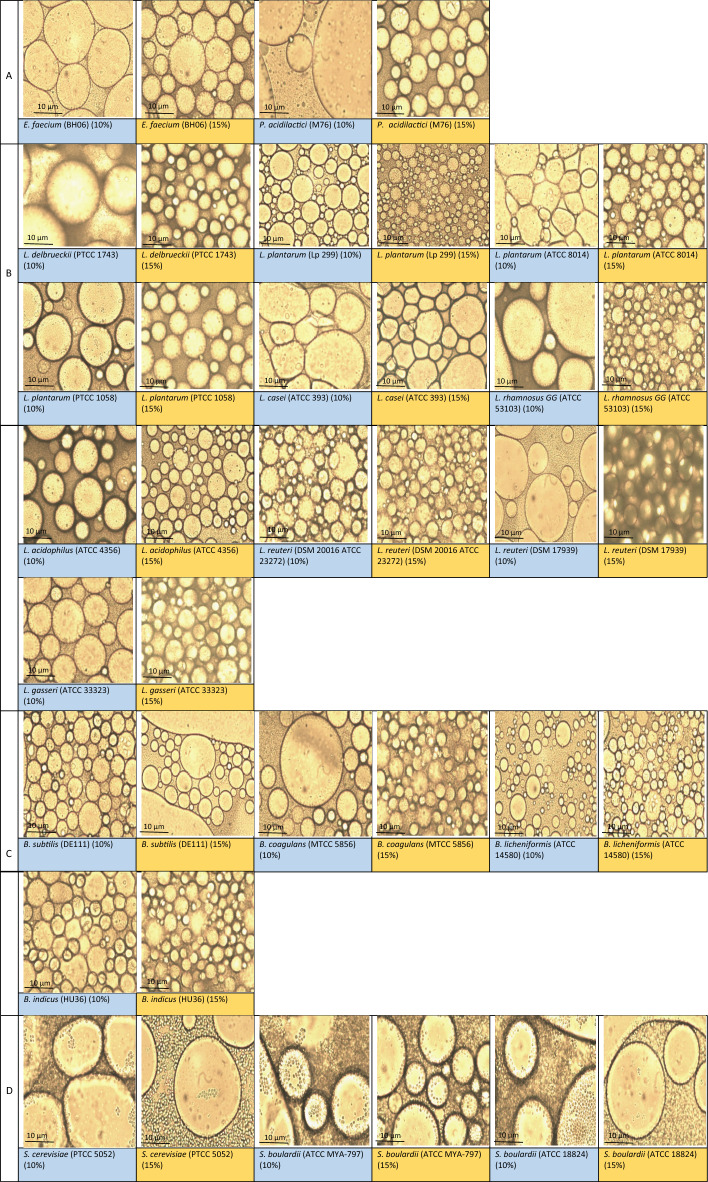
Optical micrographs of microorganisms-stabilized Pickering emulsions (Magnification × 40, Scale 10 µm); (**A**) Cocci cells, (**B**) Lactobacilli cells, (**C**) spore-forming Bacilli cells, and (**D**) Yeast cells.

The ability of microorganisms to adsorb to the interface and subsequent stability of PEs are of paramount importance. Figure S2 shows the photographs of PEs stabilized by various microorganisms at 10 wt% and 15 wt% concentration during storage. The physical stability index is also reported in Table 4. At 10 wt% concentration, PEs stabilized by *L. reuteri* (DSM 20016, ATCC 23272) remained stable for 12 days and those stabilized by *L. delbrueckii* (PTCC 1743), *L. acidophilus* (ATCC 4356)*, B. coagulans* (MTCC 5856)*,* and *B. licheniformis* (ATCC 14580) remained physically stable for 7 days. Generally, the differences in physical stability can be attributed to microorganisms’ properties (e.g., surface charge, shape, size, functional groups at the cell wall, IFT reduction, wettability and etc.)^11^. A portion of observed stability can be rooted in the ability of microorganisms in the production of surface-bound biosurfactants prior to inactivation. High molecular weight biosurfactants are efficient at O/W emulsion stabilization, whereas, low molecular weight ones are useful in IFT reduction^55^. Increasing the Pickering cells concentration (i.e., 15 wt%) significantly increased the stability of PEs (to a maximum of 35 days by *L. acidophilus* (ATCC 4356)) mainly as the result of better interface coverage. PEs formulated by 15 wt% of *B. licheniformis* (ATCC 14580)*, E. faecium* (BH06)*, L. acidophilus* (ATCC 4356)*, L. reuteri* (DSM 17939)*, L. reuteri* (DSM 20016, ATCC 23272)*, L. delbrueckii* (PTCC 1743)*, L. plantarum* (Lp 299)*, L. rhamnosus GG* (ATCC 53103)*,* and *L. gasseri* (ATCC 33323) revealed physical stability index of 100% until the end of storage (10 days) and even more (Table 4). At lower concentration, the formation of larger oil droplets (Fig. 3) led to a higher physical instability in PEs. Moreover, increasing the microorganisms’ concentration could increase the physical stability by increasing the steric hindrance around the oil droplets, reducing the bulk aqueous phase volume, more IFT reduction and higher cells adsorption at the interface (i.e., by hydrophobic, Lewis acid–base and electrostatic interactions under the effects of chemical composition and conformation of proteins, polypeptides and polysaccharides at the cell wall)^23,56,57^. The ability of microorganisms to form PEs was also confirmed by measuring the EAI. As reported in Table 3, a significant (*p* < 0.05) increase in EAI was observed by increasing the cells concentration in emulsions.

**Table 4:** Physical stability index of Pickering emulsions stabilized by 10% (left columns) or 15% (right columns) of microorganisms over time.

Names of microorganisms /group	MC (%)	Days						Obvious aqueous phase separation after—days	MC (%)	Days						Obvious aqueous phase separation after—days
		0	1	2	3	4	10			0	1	2	3	4	10	
Cocci group																
*E. faecium (*BH06)	10%	100	100	88.2	82.3	82.3	82.3	2	15%	100	100	100	100	100	100	20
*P. acidilactici* (M76)	10%	75	75	54.5	31.8	31.8	31.8	Production	15%	100	100	100	100	100	60	7
Lactobacilli group																
*L. delbrueckii* (PTCC 1743)	10%	100	100	100	100	100	87.5	7	15%	100	100	100	100	100	100	14
*L. plantarum* (Lp 299)	10%	100	83.3	83.3	73.6	73.6	73.6	1	15%	100	100	100	100	100	100	35
*L. plantarum* (ATCC 8014)	10%	100	76.4	76.4	76.4	76.4	76.4	1	15%	100	93.1	89.6	89.6	86.2	86.2	1
*L. plantarum* (PTCC 1058)	10%	100	100	100	72.2	72.2	72.2	3	15%	100	100	100	100	81.2	81.2	4
*L. casei* (ATCC 393)	10%	100	100	100	44.4	44.4	44.4	3	15%	100	100	82.3	82.3	81.2	81.2	2
*L. rhamnosus GG* (ATCC 53103)	10%	100	100	100	100	100	84.6	4	15%	100	100	100	100	100	100	10
*L. acidophilus* (ATCC 4356)	10%	100	100	100	100	100	82.3	7	15%	100	100	100	100	100	100	35
*L. reuteri* (DSM 20016, ATCC 23272)	10%	100	100	100	100	100	100	12	15%	100	100	100	100	100	100	20
*L. reuteri* (DSM 17939)	10%	100	100	78.5	78.5	78.5	78.5	2	15%	100	100	100	100	100	100	35
*L. gasseri* (ATCC 33323)	10%	100	100	86.9	86.9	86.9	85.7	2	15%	100	100	100	100	100	100	14
Spore-forming Bacilli group																
*B. subtilis* (DE111)	10%	100	68.7	68.7	66.6	66.6	66.6	1	15%	34.6	34.6	25	25	25	25	Production
*B. coagulans* (MTCC 5856)	10%	100	100	100	100	100	88.8	7	15%	100	100	100	100	88.2	88.2	4
*B. licheniformis* (ATCC 14580)	10%	100	100	100	100	100	88.8	7	15%	100	100	100	100	100	100	20
*B. indicus* (HU36)	10%	100	70	70	63.1	63.1	63.1	1	15%	100	100	80	80	72.2	72.2	2
Yeast group																
*S. cerevisiae* (PTCC 5052)	10%	61.5	61.5	61.5	61.5	61.5	61.5	Production	15%	89.2	89.2	84	84	71.4	71.4	Production
*S. boulardii* (ATCC MYA-797)	10%	62.5	56	56	41.6	41.6	41.6	Production	15%	100	100	92.8	92.8	86.6	86.6	2
*S. boulardii* (ATCC 18824)	10%	63.6	63.6	63.6	63.6	63.6	63.6	Production	15%	76.1	76.1	64	64	64	64	Production

*MC* microorganism concentration (%).

In general, the yeast cells (likely due to the presence of too much fibrous polysaccharides in the cell wall) and some bacilli species showed low tendency to stabilize PEs. However, lactobacilli species showed high PE stability likely due to a better balance between the hydrophilic and hydrophobic characters of cell wall arising from peptidoglycans (up to 70 wt%), lipoteichoic acids bound to membrane, and glycoproteinaceous materials^58–60^. The higher emulsifying ability of lactobacilli cells than cocci cells can be ascribed to the presence of pili (protruded proteinaceous surface structures with a diameter of 1 to10 nm and length 1 μm) on the lactobacilli cells and pili’s effect on cell adhesion to the O/W interface. Pili also has a significant contribution on the formation of biofilms^61^. Regarding interspecies variations, S-layer proteins (40–200 kDa, 5–15 nm thickness with highly stable conformation) are the main surface proteins in many lactobacillus species such as *L. acidophilus*, *L. casei*, and *L. rhamnosus*, but not in *L. gasseri*^60,62^. They are composed of protein or glycoprotein subunits. These proteins are bound to the cell wall by non-covalent linkages and accumulate into the surface layers. S-layer proteins of lactobacilli have a large (≈ 30%) fraction of hydrophobic amino acid residues^63^. Unlike most other bacteria, the S-layer proteins in lactobacilli are highly basic and thus have high isoelectric point values. The yeasts’ cell walls are rigid and composed of mannose, chitin and glucan. *S. cerevisiae* reveals 99% genetic similarity to *S. boulardii*^64^. These are likely the reasons for the low stability of yeast-stabilized PEs as well as not significant differences in the PE stability between *S. cerevisiae* (PTCC 5052) and *S. boulardii* (ATCC 18824). Madivala et al. (2009) studied the effect of hematite particle shape (with aspect ratios ranging from 1 to 9) on the physical stability of emulsions against coalescence^65^. They found that, the shape characteristics of particles had a dominant effect on physical stability of O/W and W/O emulsions. The emulsions prepared by elongated (high-aspect ratio) particles were more stable than those prepared by spherical (low-aspect ratio) particles of similar wettability. In this study, *L. rhamnosus GG* (ATCC 53103) as a rod-shaped bacterium and *E. faecium* (BH06) as a spherical-shaped bacterium had similar *θ*_ow_ values (75.16° ± 2.45 and 78.68° ± 0.31, respectively). However, the PEs stabilized by *L. rhamnosus GG* (ATCC 53103) particularly at lower microorganism concentration (10 wt%) was more stable than those stabilized by *E. faecium* (BH06), which were 4 and 2 days, respectively. Those behaviors were rotted in the interfacial shear rheology under the effect of surface coverage and shape-induced attractive capillary interactions. Due to a larger surface area, elongated particles have a better surface coverage at the O/W interface, which can contribute to the higher physical stability. As illustrated in Fig. S2, the overt sign of physical instability in PEs was the separation of aqueous phase at the bottom and in rare cases oiling off (e.g., *B. subtilis* (DE111), *S. cerevisiae* (PTCC 5052) and both strains of *S. boulardii*). Since the emulsion becomes cloudy after gentle stirring, creaming or flocculation are not considered as the important signs of emulsion instability, provided the oil droplets are not being subjected to coalescence^7^. Dorobantu et al., (2004) reported the formation of O/W and W/O PEs by hydrocarbon degrading bacteria. Partial hydrophobic character resulted in optimum emulsion stability^19^. In another study, Firoozmand and Rousseau reported that *S. cerevisiae* resulted in the highest emulsion stability, followed by *L. acidophilus* and *S. thermophiles*^11^. The long-term emulsion stability (4 months) reported by Firoozmand and Rousseau compared to that reported in the current study (maximum of 35 days) can be attributed to the strain-dependent characteristic of microorganisms and more importantly to the higher φ (0.8 vs. 0.5 in this study). Increasing φ increases the viscoelastic properties of emulsions and thus improves the storage stability.

The stability of food emulsions against syneresis can be considered by "freeze–thaw stability" assay. In this study, three PEs including those stabilized by 15 wt% *E. faecium* (BH06)*, 15 wt% L. delbrueckii* (PTCC 1743)*,* and 15 wt% *B. licheniformis* (ATCC 14580) were subjected to four cycles of freezing and thawing. From the third cycle, a limited syneresis (3.5%, 4.5%, and 4.8%, respectively) was observed in PEs. At fourth cycle, the syneresis significantly increased to 37.5%, 40.54%, and 45.45%, respectively. The increase in syneresis could be attributed to the structural breakdown mediated by centrifugal forces^66^.

The microstructure of PEs is shown in Fig. 3. Spherical or polyhedral oil droplets were observed in various PEs. For a same type of microorganism, smaller and more uniform oil droplets were observed by increasing the concentration of microorganism. This morphology led to a higher physical stability during storage. The adsorption of microorganisms to the interface (particularly for the yeast cells because of the larger size) was also seen in the optical micrographs.

#### Emulsion stability from a thermodynamic viewpoint

To develop stable emulsions, Δ*G*_d_ should be greater than the thermal energy of particles^67^. As reported in Table 2, *B. coagulans* (MTCC 5856) and *L. plantarum* (ATCC 8014) had the highest (55 × 10^−15^ J) and the lowest (0.06 × 10^−15^ J) values of Δ*G*_d_, respectively. Large amount of Δ*G*_d_ means that a high energy is required for the desorption of Pickering cells from the interface and therefore, the resultant PE has appropriate stability. Generally, the emulsifying capability of particulate structures depends on the particle size, IFT and *θ*_ow_. Smaller Pickering particles have usually fast kinetics of adsorption; however, the Pickering functionality is mostly influenced by the particle size (*r*^2^). For some microorganisms with low ability to reduce IFT, *θ*_ow_ and particle size contributed to increase Δ*G*_d_. Δ*G*_d_ tends to zero in the presence of highly hydrophilic (Cos 0° = 1) and hydrophobic (Cos 180° = − 1) particles. It is increased by increasing the *θ*_ow_ to 90° (Cos 90° = 0). In other words, the highest amount of irreversible attachment is observed at *θ*_ow_ values around 90°. According to Binks and Horozov (2006), Δ*G*_d_ values of rod-shaped particles are more than the Δ*G*_d_ values of spherical-shaped particles, mainly due to a favorable planar orientation of rodlike particles at the interface^1^. Δ*G*_d_ reduction upon the interfacial attachment of rodlike particles is greater than that of spherical-shaped particles, which results in a higher thermodynamic stability. Δ*G*_d_ determines the equilibrium in microorganism position at the interface and in the bulk. An activation energy is required for the detachment of particles from the interface. Larger particle size favors slower kinetics of adsorption and higher energy hurdles at the interface^1^. Therefore, the energy required for the detachment of particles from the interface rapidly decreases with decreasing particle size. As a result, the long-term physical stability of PEs stabilized by micron-sized particles is higher than that stabilized by nanoparticles^68^. The Δ*G*_d_ of rod-shaped particles depends on the aspect ratio and *θ*_ow_. At a fixed particle volume, the parallel orientation results in the stronger adsorption of rod-shaped particles with rounded ends than spherical particles to the interface and thus larger Δ*G*_d_ values^68^. As can be seen, the average Δ*G*_d_ values of lactobacilli and *Bacillus* spp. cells were higher than those of cocci cells. Generally, most PEs with high Δ*G*_d_ values showed high physical stability against coalescence during storage. However, in some PEs (e.g., those stabilized by *L. reuteri* (DSM 17939) and *E. faecium* (BH06)), despite the low amount of Δ*G*_d_, appropriate physical stability was observed over time, that could be arisen from the effect of rheological properties on the emulsion stability.

#### Rheological properties of PE

##### Apparent viscosity

Viscosity is influenced by the structural alterations due to the aggregation of emulsion droplets. Changes in the *η* of PEs as a function of shear rate (γ: 0.1–100 s^−1^) are shown in Fig. S3A–D. It was reduced by increasing the shear rate confirming shear-thinning or pseudoplastic behavior of emulsions. This behavior was ascribed to shear-induced deflocculation of oil droplets^69,70^. The *η* values of PEs stabilized by lactobacilli and *Bacillus* spp. microorganisms were greater than those stabilized by cocci and yeast cells. As reported in Table 3, the *η* of PEs at 57.6 s^−1^ ranged from 23.8 to 340.3 mPa s by 10 wt% of *L. plantarum* (ATCC 8014) and *L. reuteri* (DSM 20016, ATCC 23272), respectively, and from 43.3 to 1094.9 mPa.s by 15 wt% of *S. boulardii* (ATCC MYA-797) and *L. reuteri* (DSM 17939), respectively. Therefore, the viscosity of PEs can be adjusted by appropriate selection of microorganism and its concentration. The variations in the *η* might be attributed to the strength of interactions between cell-stabilized oil droplets, shape and size of microorganism, and also the presence of cell-bound exopolysaccharides in the continuous phase. A pseudoplastic behavior in PEs stabilized by microorganisms or other Pickering particles was similarly reported by Firoozmand and Rousseau and Boostani et al.^11,70^. Generally, the *η* of PEs was significantly increased by increasing the cells concentration likely owing to smaller oil droplet size (or larger specific surface area) and higher viscosity of continuous phase. In some cell-stabilized PEs (e.g., by *L. reuteri* (DSM 20016, ATCC 23272)), a decrease in *η* was observed by increasing the microorganism concentration. This reduction might be due to the higher concentration of free cells in the continuous phase that could reduce the friction via “ball-bearing” mechanism^71^. Particles with a spherical and smooth surface can highly reduce the friction as a result of considerable rolling.

The analysis results of four rheological models are reported in Table 5. Higher coefficient of determination *(R*^2^) and lower root mean square error (RMSE) values indicate a better fitting. Accordingly, Herschel-Bulkely model was better than Power Law, Casson and Bingham models to describe flow behavior of PEs. The flow behavior index values (*n* < 1) confirmed the pseudoplasticity. The values ranged from 0.79 to 0.26 for PEs stabilized by 10 wt% of *L. plantarum* (ATCC 8014) and *L. reuteri* (DSM 17939), respectively and from 0.83 to 0.49 for those stabilized by 15 wt% of *P. acidilactici* (M76) and *L. reuteri* (DSM 17939), respectively. For a same cell, an increase in the concentration generally increased the pseudoplasticity (i.e., lower *n* values) and consistency index (*k* or structuration degree) of PEs. The yield stress (*τ*_0_) of cell-stabilized PEs was dependent on microorganism type and concentration.

**Table 5:** Rheological analysis of 4 different models (Power Law, Herschel-Bulkley, Bingham, and Casson) in Pickering emulsions stabilized by 10% and 15% of microorganism cells.

MO Names	Power law				Bingham				Herschel Bulkley					Casson			
	K (Pa.s^n^)	n	R^2^ (%)	RMSE	µ (Pa s)	τ_0_ (Pa)	R^2^ (%)	RMSE	K (Pa s^n^)	n	τ_0_ (Pa)	R^2^ (%)	RMSE	k (Pa s^0.5^)	τ_0_ (Pa)	R^2^ (%)	RMSE
A 10%	0.28 ± 0.03^k^	0.51 ± 0.02^c^	95.90^h^	0.16^ij^	0.03 ± 9.31^ef^	0.41 ± 0.02^k^	97.81^b^	0.03^h^	0.08 ± 0.01^jk^	0.77 ± 0.03^c^	0.31 ± 0.02^i^	99.06^f^	0.04^g^	0.13 ± 0.12^def^	0.70 ± 0.08^i^	98.82^a^	0.44^j^
A 15%	0.92 ± 0.04^I^*	0.63 ± 0.01^C^*	99.63^C^*	0.31^H^*	0.17 ± 0.00^EF^*	1.36 ± 0.17^H^*	97.51^E^	0.28^H^*	0.67 ± 0.03^H^*	0.70 ± 0.01^C^	0.49 ± 0.05^G^*	99.91^D^	0.04^I^	0.36 ± 0.05^C^*	0.85 ± 0.17^F^*	99.33^C^*	4.33^H^*
B 10%	Unstable																
B 15%	2.88 ± 0.03^D^	0.63 ± 0.02^C^	97.79^I^	0.21^I^	0.05 ± 0.00^JK^	0.49 ± 0.03^N^	99.14^A^	0.05^M^	0.11 ± 0.00^KL^	0.83 ± 0.01^B^	0.37 ± 0.02^H^	99.81^E^	0.06^H^	0.18 ± 0.04^EF^	0.72 ± 0.23^G^	99.74^A^	0.94^L^
C 10%	3.68 ± 0.17^c^*	0.25 ± 0.01^ef^	94.63^j^	0.71^c^*	0.11 ± 0.00^b^	4.02 ± 0.18^c^*	92.82^i^	0.41^a^	0.89 ± 0.11^e^	0.53 ± 0.02^g^	2.88 ± 0.14^c^*	99.13^e^	0.15^c^	0.20 ± 0.17^de^	1.32 ± 0.05^c^*	97.89^c^	4.19^c^
C 15%	2.79 ± 0.10^E^	0.45 ± 0.01^G^*	99.32^E^*	0.53^E^	0.23 ± 0.01^C^*	3.59 ± 0.35^D^	94.10^K^*	0.71^C^*	1.70 ± 0.04^C^*	0.55 ± 0.00^G^	1.39 ± 0.05^E^	99.96^B^	0.16^F^	0.37 ± 0.24^C^*	1.18 ± 0.05^CD^	98.33^I^*	6.94^C^*
D 10%	0.14 ± 0.00^l^	0.67 ± 0.01^a^*	99.56^b^	0.07^m^	0.03 ± 0.00^ef^	0.17 ± 0.04^m^	96.10^e^*	0.03^h^	0.16 ± 0.01^i^	0.65 ± 0.02^e^*	0.00 ± 0.02^l^	99.45^c^	0.00^i^	0.18 ± 0.05^bcd^	0.37 ± 0.2^l^	96.08^h^*	0.38^l^
D 15%	1.10 ± 0.05^H^*	0.57 ± 0.01^D^	99.39^D^	0.33^GH^*	0.16 ± 0.00^F^*	1.42 ± 0.23^G^*	94.68^I^	0.36^F^*	1.08 ± 0.12^E^*	0.57 ± 0.02^F^	0.01 ± 0.16^N^	99.39^H^	0.23^D^*	0.36 ± 0.14^C^*	0.80 ± 0.23^G^*	94.41^N^	3.96^I^*
E 10%	0.13 ± 0.01^l^	0.59 ± 0.03^b^	96.96^g^	0.09^l^	0.02 ± 0.00^f^	0.21 ± 0.01^l^	97.88^a^*	0.02^h^	0.05 ± 0.00^jk^	0.79 ± 0.04^b^*	0.15 ± 0.02^jk^	98.84^h^	0.04^g^	0.11 ± 0.17^ef^	0.58 ± 0.09^j^	98.42^b^	0.27^n^
E 15%	0.26 ± 0.02^M^*	0.56 ± 0.02^D^	98.41^G^*	0.12^J^*	0.03 ± 0.00^K^	0.37 ± 0.03^O^*	96.88^F^	0.09^L^	0.14 ± 0.01^K^*	0.69 ± 0.02^CD^	0.19 ± 0.03^J^	99.25^J^*	0.04^I^	0.15 ± 0.05^F^	0.65 ± 0.14^H^*	98.22^K^	0.52^M^*
F 10%	2.32 ± 0.16^e^*	0.27 ± 0.03^e^	95.68^i^*	0.28^f^*	0.11 ± 0.00^b^*	2.43 ± 0.18^f^*	91.35^l^	0.18^d^*	1.10 ± 0.07^d^*	0.68 ± 0.02^d^	2.22 ± 0.11^d^*	98.93^g^*	0.05^g^	0.20 ± 0.12^de^*	1.15 ± 0.08^e^*	95.60^i^*	2.50^g^*
F 15%	0.41 ± 0.04^L^	0.45 ± 0.03^G^*	93.73^N^	0.22^I^	0.03 ± 0.00^K^	0.54 ± 0.05^M^	93.92^M^*	0.08^L^	0.13 ± 0.04^KL^	0.68 ± 0.07^D^	0.37 ± 0.07^H^	95.93^M^	5.50^A^*	0.13 ± 0.04^F^	0.75 ± 0.17^G^	92.56^O^	0.55^M^
G 10%	0.70 ± 0.04^i^	0.24 ± 0.01^ef^	90.15^k^	0.17^i^	0.02 ± 9.14^f^	0.75 ± 0.02^h^	95.38^f^	0.04^gh^	0.09 ± 0.02^j^	0.66 ± 0.05^e^	0.64 ± 0.03^g^*	97.75^j^	0.04^g^	0.08 ± 0.30^f^	0.87 ± 0.10^g^	97.74^d^	0.31^m^
G 15%	0.93 ± 0.03^I^*	0.56 ± 0.00^D^*	99.71^B^*	0.21^I^*	0.13 ± 0.00^G^*	1.29 ± 0.16^I^*	95.95^G^	0.32^G^*	0.70 ± 0.02^H^*	0.62 ± 0.00^E^	0.36 ± 0.04^HI^	99.92^CD^*	0.07^H^	0.30 ± 0.11^D^*	0.86 ± 0.30^F^	98.39^G^*	3.09^J^*
H 10%	2.10 ± 0.04^g^	0.35 ± 0.00^d^	99.66^a^*	0.19^h^	0.10 ± 0.00^b^	2.43 ± 0.24^f^	86.87^m^	0.38^ab^	1.93 ± 0.14^b^	0.37 ± 0.01^i^	0.18 ± 0.15^j^	99.68^b^*	0.07^f^	0.24 ± 0.18^ab^	1.09 ± 0.20^f^	93.46^j^	3.11^f^
H 15%	9.73 ± 0.78^B^*	0.34 ± 0.02^I^	92.66^O^	3.21^A^*	0.44 ± 0.02^B^*	11.69 ± 0.75^B^*	93.03^N^*	1.39^B^*	2.50 ± 0.51^B^*	0.61 ± 0.04^E^*	8.38 ± 0.75^B^*	98.11^K^	0.54^C^*	0.44 ± 0.21^B^*	1.69 ± 0.20^B^*	96.73^M^*	18.13^B^*
I 10%	0.33 ± 0.01^k^	0.62 ± 0.01^b^	99.58^b^	0.11^k^	0.06 ± 0.00^d^	0.46 ± 0.06^j^	96.82^c^	0.12^ef^	0.26 ± 0.02^h^	0.66 ± 0.01^e^	0.11 ± 0.03^k^	99.69^b^	0.09^e^	0.22 ± 0.05^abc^	0.62 ± 0.20^ij^	97.12^f^	1.06^h^
I 15%	0.59 ± 0.00^K^*	0.77 ± 0.00^A^*	99.97^A^	0.20^J^*	0.21 ± 0.00^D^*	0.77 ± 0.14^L^*	98.83^B^*	0.19^I^	0.56 ± 0.00^I^*	0.78 ± 0.00^A^*	0.11 ± 0.02^L^	99.99^A^	0.11^G^	0.43 ± 0.08^B^*	0.63 ± 0.12^H^	99.61^B^*	5.10^G^*
J 10%	7.10 ± 0.63^a^*	0.22 ± 0.02^fg^	74.71^m^	2.51^a^*	0.18 ± 0.00^a^	7.57 ± 0.27^a^*	94.57^g^	0.40^b^	0.42 ± 0.15^g^	0.81 ± 0.08^a^*	7.10 ± 0.37^a^*	95.59^m^	0.38^a^*	0.23 ± 0.17^abc^	1.57 ± 0.40^a^*	89.65^m^	8.57^a^*
J 15%	1.86 ± 0.12^G^	0.49 ± 0.01^F^*	98.29^H^*	0.64^D^	0.18 ± 0.00^E^	2.56 ± 0.26^F^	94.84^H^	0.52^D^*	1.00 ± 0.11^F^*	0.62 ± 0.02^E^	1.24 ± 0.17^F^	99.41^G^*	0.11^G^	0.33 ± 0.03^C^*	1.09 ± 0.26^E^	99.03^D^*	5.16^F^
K 10%	5.25 ± 0.09^b^	0.19 ± 0.00^g^	98.03^e^*	0.38^d^	0.09 ± 0.00^c^	5.55 ± 0.29^b^	80.10^o^	0.40^bc^	3.26 ± 0.58^a^	0.26 ± 0.03^k^	1.95 ± 0.58^e^	98.44^i^	0.18^b^	0.17 ± 0.20^cde^	1.45 ± 0.25^b^	91.83^k^	5.02^b^
K 15%	16.87 ± 0.75^A^*	0.32 ± 0.01^J^*	97.35^K^	3.04^B^*	0.69 ± 0.04^A^*	19.57 ± 1.34^A^*	90.99^P^*	2.33^A^*	6.84 ± 0.58^A^*	0.49 ± 0.01^H^*	10.91 ± 0.7^A^*	99.61^F^*	0.63^B^*	0.56 ± 0.20^A^*	1.91 ± 0.30^A^*	98.17^L^*	30.63^A^*
L 10%	0.73 ± 0.03^h^	0.37 ± 0.01^d^*	98.35^d^*	0.15^j^	0.04 ± 0.00^e^	0.88 ± 0.07^g^	92.46^j^	0.11^ef^	0.39 ± 0.04^g^	0.50 ± 0.02^h^	0.38 ± 0.05^h^	99.25^d^	0.07^f^	0.14 ± 0.31^def^	0.86 ± 0.25^g^	96.69^g^	0.79^i^
L 15%	2.62 ± 0.10^F^*	0.29 ± 0.01^K^	97.04^L^	0.42^F^*	0.09 ± 0.00^H^	2.95 ± 0.16^E^*	92.24^O^	0.28^H^*	0.88 ± 0.03^G^*	0.50 ± 0.00^H^	1.83 ± 0.03^D^*	99.93^C^	0.07^H^	0.19 ± 0.20^EF^	1.20 ± 0.18^D^*	98.75^E^*	3.09^J^*
M 10%	0.49 ± 0.06^j^	0.35 ± 0.03^d^	87.03^l^	0.25^g^	0.02 ± 0.00^f^	0.55 ± 0.07^i^	80.35^n^	0.13^e^	0.49 ± 0.25^f^	0.35 ± 0.10^j^	0.00 ± 0.26^l^	87.03^n^	0.11^d^	0.12 ± 0.20^def^	0.73 ± 0.17^h^	74.23^o^	0.42^k^
M 15%	Unstable																
N 10%	2.25 ± 0.07^f^	0.38 ± 0.00^d^	99.02^c^*	0.34^e^	0.12 ± 0.00^b^	2.71 ± 0.22^e^	92.27^k^	0.33^c^	1.27 ± 0.07^c^*	0.49 ± 0.01^h^	1.11 ± 0.08^f^	99.83^a^	0.14^c^	0.26 ± 0.04^a^	1.13 ± 0.07^ef^	97.25^e^	3.84^d^
N 15%	2.98 ± 0.17^C^*	0.38 ± 0.01^H^	97.43^J^	0.77^C^*	0.17 ± 0.00^EF^	3.64 ± 0.25^C^*	94.62^J^*	0.36^F^	1.19 ± 0.13^D^	0.57 ± 0.02^F^*	2.11 ± 0.18^C^*	99.36^I^	0.24^D^*	0.30 ± 0.13^D^	1.23 ± 0.15^C^*	98.36^H^*	5.57^E^*
O 10%	2.57 ± 0.40^d^*	0.30 ± 0.04^ef^	68.20^n^	1.66^b^*	0.11 ± 0.00^b^	3.16 ± 0.12^d^*	96.76^d^	0.08^fg^	0.04 ± 0.01^k^	0.68 ± 0.08^d^	3.34 ± 0.12^b^*	97.52^l^	0.38^a^*	0.18 ± 0.23^bcd^	1.27 ± 0.09^d^*	84.53^n^	3.69^e^
O 15%	0.92 ± 0.01^I^	0.68 ± 0.00^B^*	99.96^A^*	0.36^G^	0.23 ± 0.00^C^*	1.22 ± 0.22^J^	97.57^D^*	0.41^E^*	0.89 ± 0.02^G^*	0.69 ± 0.00^CD^	0.07 ± 0.04^M^	99.97^B^*	0.19^E^	0.45 ± 0.12^B^*	0.72 ± 0.09^G^	98.29^J^*	5.66^D^*
P 10%	0.16 ± 0.01^l^	0.54 ± 0.02^c^	97.68^f^*	0.09^l^	0.02 ± 0.00^f^	0.21 ± 0.03^l^	93.23^h^	0.04^gh^	0.15 ± 0.03^i^	0.56 ± 0.04^f^	0.02 ± 0.04^l^	97.71^k^	0.02^h^	0.13 ± 0.20^def^	0.51 ± 0.04^k^	91.17^l^*	0.23^o^
P 15%	0.66 ± 0.07^J^*	0.51 ± 0.02^E^	96.60^M^	0.37^G^*	0.07 ± 0.00^I^	0.88 ± 0.11^K^*	93.99^L^	0.15^J^*	0.41 ± 0.10^J^*	0.61 ± 0.05^E^	0.35 ± 0.15^I^*	97.03^L^	0.16^F^*	0.22 ± 0.07^DE^*	0.78 ± 0.12^G^*	90.98^P^	1.56^K^*
Q 10%	Unstable																
Q 15%	Unstable																
R 10%	Unstable																
R 15%	0.15 ± 0.01^N^	0.68 ± 0.02^B^	98.51^F^	0.13^J^	0.03 ± 8.61^K^	0.26 ± 0.02^P^	98.73^C^	0.12^K^	0.08 ± 0.01^L^	0.82 ± 0.02^B^	0.17 ± 0.02^K^	99.42^G^	0.04^I^	0.16 ± 0.17^F^	0.57 ± 0.06^I^	98.41^F^	0.53^M^
S 10%	Unstable																
S 15%	Unstable																

Pickering emulsions which remained stable after 1 day were only analyzed.

Different lowercase and uppercase letters indicate significant differences (*p* < 0.05) among different microorganisms at the same concentration of 10 wt% and 15 wt%, respectively. For a same microorganism, the asterisk (*) shows significant differences (*p* < 0.05) between different concentrations (10 wt% and 15 wt%). (A):* E. faecium* (BH06); (B): *P. acidilactici* (M76); (C): *L. delbrueckii* (PTCC 1743); (D): *L. plantarum* (Lp 299); (E): *L. plantarum* (ATCC 8014); (F): *L. plantarum* (PTCC 1058); (G): *L. casei* (ATCC 393); (H): *L. rhamnosus GG* (ATCC 53103); (I): *L. acidophilus* (ATCC 4356); (J): *L. reuteri* (DSM 20016, ATCC 23272); (K): *L. reuteri* (DSM 17939); (L): *L. gasseri* (ATCC 33323); (M): *B. subtilis* (DE111); (N): *B. coagulans* (MTCC 5856); (O): *B. licheniformis* (ATCC 14580); (P): *B. indicus* (HU36); (Q): *S. cerevisiae* (PTCC 5052); (R): *S. boulardii* (ATCC MYA-797); (S): *S. boulardii* (ATCC 18824).

##### Dynamic rheological properties

**Figure 4:**
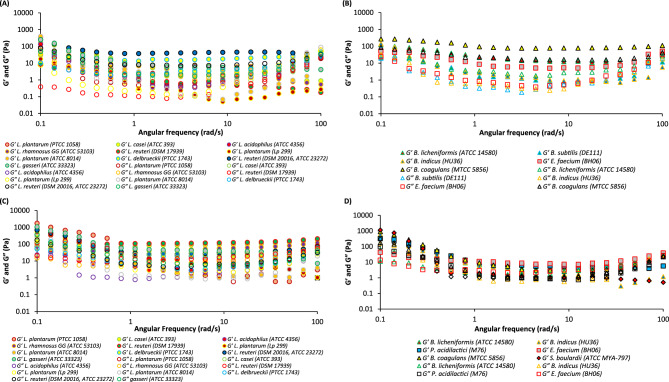
Frequency sweep test results of Pickering emulsions stabilized by (**A**) 10% of lactobacilli microorganisms, (**B**) 10% of spore-forming bacillus spp., cocci and yeast cells, (**C**) 15% of lactobacilli cells and (**D**) 15% of spore-forming bacillus spp. cocci and yeast cells; The samples which remained physically stable after 24 h were only analyzed.

The stability and application of PEs are determined by the viscoelastic properties^72^. Figure S4 illustrates the results of amplitude sweep test. The elastic modulus (*G*′) was greater than the viscous modulus (*G*″), indicating a higher degree of rigidity. The critical strain values were dependent on the microorganisms’ type and concentration. However, for most samples, the critical strain values were below 10% (Table S2). As can be seen, the strain value of 0.1% was within the LVE region of various PEs. Therefore, this value was selected for frequency sweep test. The results of frequency sweep test are shown in Fig. 4. Most PEs stabilized by different microorganisms showed dominant elastic properties (i.e., *G*' > *G*″) indicating the formation of 3D networks resulting in the high physical stability of emulsions during storage (section "Droplet size and droplet charge"). The dominant elastic properties could be related to the inter-droplet interactions of oil droplets as a result of cells’ packing at the interface^25,73^. Similar rheological properties were reported by Boostani et al. and Lu et al.^3,70^. The dependency of *Gʹ* and *Gʺ* to frequency was related to the type of Pickering microorganism. An increase in cell concentration increased the viscoelastic properties of PEs. The highest *Gʹ* values of PEs at the studied frequency range were measured in the samples stabilized by 10 wt% of *B. coagulans* (MTCC 5856)*, L. reuteri* (DSM 20016, ATCC 23272)*,* and *L. delbrueckii* (PTCC 1743) and 15 wt% of *L. reuteri* (DSM 20016, ATCC 23272)*, L. reuteri* (DSM 17939)*, L. delbrueckii* (PTCC 1743)*,* and *L. rhamnosus GG* (ATCC 53103). The loss factor results (i.e., the ratio of *G''*/*G'* or tan *δ*) are reported in Fig. S5. The values < 1 and > 1 indicate dominant elastic and dominant viscous characters, respectively. For the most PE samples, no crossover (*G''* = *G'*) was observed over the studied range. For some samples stabilized by *L. plantarum* (Lp 299), *L. gasseri* (ATCC 33323), *L. casei* (ATCC 393), *L. rhamnosus GG* (ATCC 53103), *L. plantarum* (ATCC 8014), *L. reuteri* (DSM 17939), *S. boulardii* (ATCC MYA-797), *B. subtilis* (DE111), and *B. indicus* (HU36), a crossover was observed at higher frequency values indicating the change from a predominant elastic character to viscous character. Despite the dominant elastic behavior, PEs were not real gels (0.1 < tan *δ*)^27^. The *η* results were also confirmed by complex viscosity (η*) results (Fig. S6). The decrease in η* by increasing the frequency was attributed to droplet deflocculation, network disentanglement and breakdown of junction zones.

## Conclusion

This study showed that probiotic microorganisms such as lactobacillus, *Bacillus* spp. and coccus bacteria are able to stabilize PEs at 10 wt% and 15 wt% concentrations. Increasing the cells concentration resulted in a significant increase of physical stability against droplet coalescence and aqueous phase separation from a maximum of 12 days to 35 days. *L. reuteri* (DSM 20016, ATCC 23272), *L. reuteri* (DSM 17939), *L. acidophilus* (ATCC 4356), *L. plantarum* (Lp 299), *E. faecium* (BH06), and *B. licheniformis* (ATCC 14580) revealed a high emulsifying ability. Pickering functionality of probiotic microorganisms depended on the shape, size, and charge of cells, IFT reduction, *θ*_ow_ and possibly cell wall composition. Pseudoplastic and predominant elastic behaviors were observed in PEs. Cell-stabilized PEs might have novel applications in food, drug and cosmetics industries. The stability of PEs under the formation of biopolymer/cell entangled networks, and the hydrophobic modification of cell wall is under research.

### Supplementary Information

Supplementary Information 1.

Supplementary Information 2.

## Data Availability

The authors confirm that the data supporting the findings of this study are available within the article and its supplementary materials.

## Abbreviations

ANOVA: Analysis of variance
*D*_4,3_: Volume-weighted average droplet size
DDW: Double distilled water
DLS: Dynamic light scattering
Δ*G*_d_: The free energy of detachment
EAI: Emulsion activity index
EPS: Extracellular polysaccharides
*η*: Apparent viscosity
*η**: Complex viscosity
φ: The volume fraction of dispersed phase
*G*′: Elastic modulus
*G*″: Viscous modulus
IFT: Interfacial tension
LCT: Long-chain triacylglycerol
LVE: Linear viscoelastic
MCT: Medium-chain triacylglycerol
MRS: De man, Rogosa and Sharpe
OD: Optical density
PBS: Phosphate buffer solution
PE: Pickering emulsion
*R*^2^: Coefficient of determination
RMSE: Root mean square error
SDB: Sabouraud dextrose broth
SEM: Scanning electron microscopy
SPAN: Droplet size distribution width
*tan δ*: Loss factor
*θ*_ow_: Three-phase contact angle
TSB: Tryptic soy broth
