# Supplementary material for: Promising application of probiotic microorganisms as Pickering emulsions stabilizers
Source: Sci Rep. 2023 Sep 23;13:15915. doi: 10.1038/s41598-023-43087-w (PMC10517997; doi:10.1038/s41598-023-43087-w)
Supplement: Supplementary file 1 — Supplementary Information 1. [file 41598_2023_43087_MOESM1_ESM.docx]

**Supplementary information for**

**Promising application of probiotic microorganisms as Pickering emulsions stabilizers**

Maryam Nejadmansouri *^a^*, Mohammad Hadi Eskandari *^a^*, Gholam Hossein Yousefi *^b^*, Masoud Riazi *^c,d^*, Seyed Mohammad Hashem Hosseini *^a,*^*

^a^ Department of Food Science and Technology, School of Agriculture, Shiraz University, Shiraz, Iran

^b^ Department of Pharmaceutics, School of Pharmacy, Shiraz University of Medical Sciences, Shiraz, Iran

^c^ Enhanced Oil Recovery (EOR) Research Centre, IOR/EOR Research Institute, Shiraz University, Shiraz, Iran

^d^ Department of Petroleum Engineering, School of Chemical and Petroleum Eng., Shiraz University, Shiraz, Iran

* Corresponding author

*E-mail address:* [hhosseini@shirazu.ac.ir](mailto:hhosseini@shirazu.ac.ir) (S. M. H. Hosseini).

**Table. S1** Particle size of microorganisms

**Table. S2** Critical strain values (determined by amplitude sweep test) of Pickering emulsions stabilized by various concentrations (10% and 15%) of microorganisms

**Fig. S1** Droplet size distribution of Pickering emulsions stabilized by (A) 10% and (B) 15% of different microorganisms

**Fig. S2** Visual observation of Pickering emulsions stabilized by 10% (I) and 15% (II) microorganisms during storage. (A): *E. faecium* (BH06); (B): *P. acidilactici* (M76); (C): *L. delbrueckii* (PTCC 1743); (D): *L. plantarum* (Lp 299); (E): *L. plantarum* (ATCC 8014); (F): *L. plantarum* (PTCC 1058); (G): *L. casei* (ATCC 393); (H): *L. rhamnosus GG* (ATCC 53103); (I): *L. acidophilus* (ATCC 4356); (J): *L. reuteri* (DSM 20016, ATCC 23272); (K): *L. reuteri* (DSM 17939); (L): *L. gasseri* (ATCC 33323); (M): *B. subtilis* (DE111); (N): *B. coagulans* (MTCC 5856); (O): *B. licheniformis* (ATCC 14580); (P): *B. indicus* (HU36); (Q) *S. cerevisiae* (PTCC 5052); (R): *S. boulardii* (ATCC MYA-797); (S): *S. boulardii* (ATCC 18824). Red arrows illustrate the sign of physical instability in Pickering emulsions.

**Fig. S3** Apparent viscosity of Pickering emulsions stabilized by (A) 10% of lactobacilli microorganisms, (B) 10% of spore-forming bacillus spp., cocci and yeast cells, (C) 15% of lactobacilli cells and (D) 15% of spore-forming bacillus spp. cocci and yeast cells; The samples which remained physically stable after 24 h were only analyzed.

**Fig. S4** Amplitude sweep test results of Pickering emulsions stabilized by (A) 10% of lactobacilli microorganisms, (B) 10% of spore-forming bacillus spp., cocci and yeast cells, (C) 15% of lactobacilli cells and (D) 15% of spore-forming bacillus spp. cocci and yeast cells; The samples which remained physically stable after 24 h were only analyzed.

**Fig. S5** Loss factor results of Pickering emulsions stabilized by (A) 10% of lactobacilli microorganisms, (B) 10% of spore-forming bacillus spp., cocci and yeast cells, (C) 15% of lactobacilli cells and (D) 15% of spore-forming bacillus spp. cocci and yeast cells; The samples which remained physically stable after 24 h were only analyzed.

**Fig. S6** Complex viscosity of Pickering emulsions stabilized by (A) 10% of lactobacilli microorganisms, (B) 10% of spore-forming bacillus spp., cocci and yeast cells, (C) 15% of lactobacilli cells and (D) 15% of spore-forming bacillus spp. cocci and yeast cells; The samples which remained physically stable after 24 h were only analyzed.

**Table S1**

| Names of microorganisms / Groups | Radius microorganism (µm) | Length microorganism (µm) |
| --- | --- | --- |
| **Cocci group (Cocci-shaped)** | | |
| *E. faecium* (BH06) | 0.34±0.03 | - |
| *P. acidilactici* (M76) | 0.40±0.03 | - |
| **Lactobacilli group (Rod-shaped)** | | |
| *L. delbrueckii* (PTCC 1743) | 0.31±0.07 | 1.23±0.24 |
| *L. plantarum* (Lp 299) | 0.30±0.01 | 1.42±0.01 |
| *L. plantarum* (ATCC 8014) | 0.30±0.01 | 1.74±0.28 |
| *L. plantarum* (PTCC 1058) | 0.23±0.01 | 1.86±0.33 |
| *L. casei* (ATCC 393) | 0.29±0.00 | 1.63±0.35 |
| *L. rhamnosus GG* (ATCC 53103) | 0.30±0.03 | 1.62±0.40 |
| *L. acidophilus* (ATCC 4356) | 0.38±0.10 | 1.90±0.66 |
| *L. reuteri* (DSM 20016, ATCC 23272) | 0.25±0.05 | 1.53±0.24 |
| *L. reuteri* (DSM 17939) | 0.32±0.04 | 1.26±0.28 |
| *L. gasseri* (ATCC 33323) | 0.29±0.01 | 1.98±0.43 |
| **Spore-forming** **Bacilli group (Rod-shaped)** | | |
| *B. subtilis* (DE111) | 0.47±0.02 | 2.62±0.56 |
| *B. coagulans* (MTCC 5856) | 0.44±0.01 | 1.58±0.37 |
| *B. licheniformis* (ATCC 14580) | 0.26±0.00 | 1.28±0.17 |
| *B. indicus* (HU36) | 0.24±0.05 | 1.62±0.58 |
| **Yeast group (Ellipsoid-shaped)** | | |
| *S. cerevisiae* (PTCC 5052) | 1.70±0.24 | - |
| *S. boulardii* (ATCC MYA-797) | 1.83±0.21 | - |
| *S. boulardii* (ATCC 18824) | 2.01±0.18 | - |

**Table S2**

| Names of microorganisms / Groups | MC (%) | Critical strain value (%) | MC (%) | Critical strain value (%) |
| --- | --- | --- | --- | --- |
| **Cocci group (Cocci-shaped)** | | | | |
| *E. faecium* (BH06) | 10 | 2.21 | 15 | 1.61 |
| *P. acidilactici* (M76) | 10 | ND | 15 | 14.9 |
| **Lactobacilli group (Rod-shaped)** | | | | |
| *L. delbrueckii* (PTCC 1743) | 10 | 4.17 | 15 | 1.17 |
| *L. plantarum* (Lp 299) | 10 | ND | 15 | 1.17 |
| *L. plantarum* (ATCC 8014) | 10 | ND | 15 | ND |
| *L. plantarum* (PTCC 1058) | 10 | 0.853 | 15 | 38.6 |
| *L. casei* (ATCC 393) | 10 | 4.17 | 15 | 20.4 |
| *L. rhamnosus GG* (ATCC 53103) | 10 | 3.04 | 15 | 2.21 |
| *L. acidophilus* (ATCC 4356) | 10 | 4.17 | 15 | 3.04 |
| *L. reuteri* (DSM 20016, ATCC 23272) | 10 | 3.04 | 15 | 2.21 |
| *L. reuteri* (DSM 17939) | 10 | 7.88 | 15 | 10.8 |
| *L. gasseri* (ATCC 33323) | 10 | 5.74 | 15 | 2.21 |
| **Spore-forming** **Bacilli group (Rod-shaped)** | | | | |
| *B. subtilis* (DE111) | 10 | ND | 15 | ND |
| *B. coagulans* (MTCC 5856) | 10 | 1.17 | 15 | 0.853 |
| *B. licheniformis* (ATCC 14580) | 10 | 28.1 | 15 | 7.88 |
| *B. indicus* (HU36) | 10 | ND | 15 | 0.853 |
| **Yeast group (Ellipsoid-shaped)** | | | | |
| *S. cerevisiae* (PTCC 5052) | 10 | ND | 15 | ND |
| *S. boulardii* (ATCC MYA-797) | 10 | ND | 15 | 0.853 |
| *S. boulardii* (ATCC 18824) | 10 | ND | 15 | ND |

ND: Not determined; MC: Microorganism concentration

| **(A)** | **(B)** |
| --- | --- |

**Fig. S1**

**(I)**

| Day | Cocci group | | Lactobacilli group | | | | | | | | | | Spore-forming Bacilli group | | | | Yeast group | | |
| --- | --- | --- | --- | --- | --- | --- | --- | --- | --- | --- | --- | --- | --- | --- | --- | --- | --- | --- | --- |
|  | A | B | C | D | E | F | G | H | I | J | K | L | M | N | O | P | Q | R | S |
| 0 | 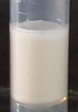 | 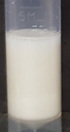 | 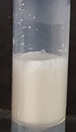 | 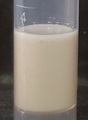 | 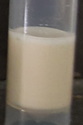 | 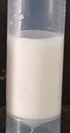 | 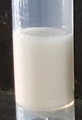 | 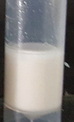 | 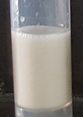 | 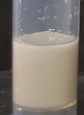 | 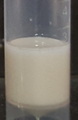 | 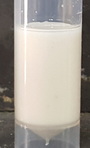 | 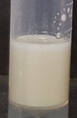 | 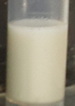 | 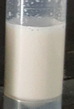 | 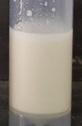 | 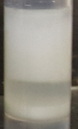 | 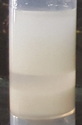 | 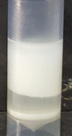 |
| 1 | 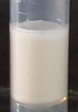 | 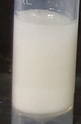 | 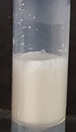 | 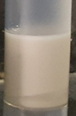 | 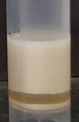 | 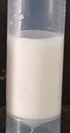 | 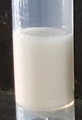 | 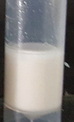 | 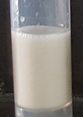 | 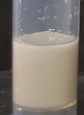 | 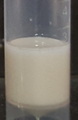 | 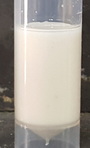 | 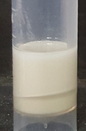 | 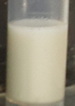 | 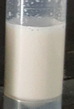 | 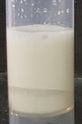 | 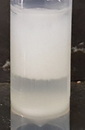 | 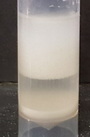 | 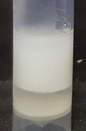 |
| 2 | 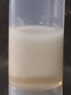 | 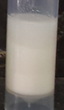 | 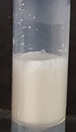 | 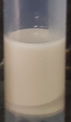 | 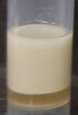 | 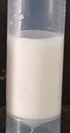 | 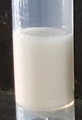 | 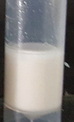 | 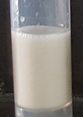 | 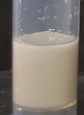 | 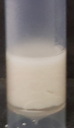 | 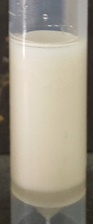 | 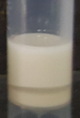 | 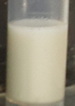 | 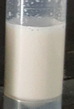 | 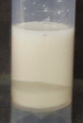 | 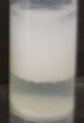 | 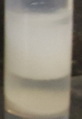 | 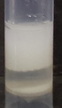 |
| 4 | 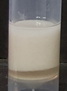 | 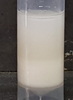 | 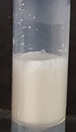 | 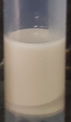 | 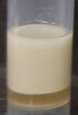 | 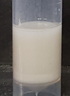 | 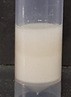 | 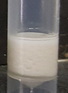 | 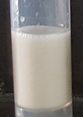 | 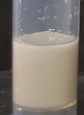 | 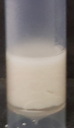 | 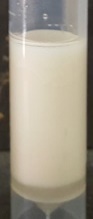 | 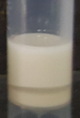 | 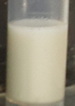 | 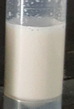 | 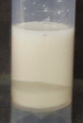 | 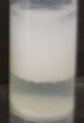 | 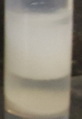 | 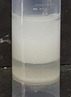 |
| 10 | 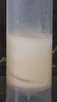 | 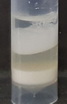 | 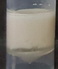 | 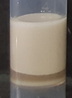 | 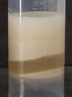 | 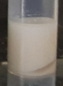 | 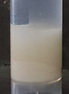 | 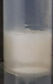 | 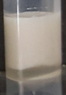 | 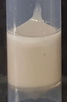 | 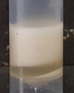 | 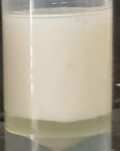 | 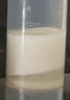 | 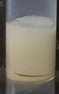 | 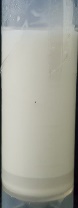 | 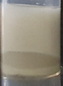 | 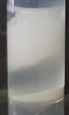 | 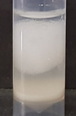 | 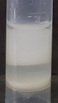 |

**(II)**

| Day | Cocci group | | Lactobacilli group | | | | | | | | | | Spore-forming Bacilli group | | | | Yeast group | | |
| --- | --- | --- | --- | --- | --- | --- | --- | --- | --- | --- | --- | --- | --- | --- | --- | --- | --- | --- | --- |
|  | A | B | C | D | E | F | G | H | I | J | K | L | M | N | O | P | Q | R | S |
| 0 | 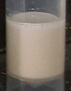 | 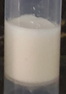 | 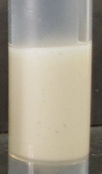 | 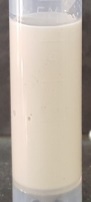 | 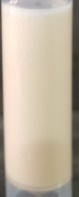 |  |  |  |  |  |  |  |  |  |  |  |  |  |  |
| 1 |  |  |  |  |  |  |  |  |  |  |  |  |  |  |  |  |  |  |  |
| 2 |  |  |  |  |  |  |  |  |  |  |  |  |  |  |  |  |  |  |  |
| 4 |  |  |  |  |  |  |  |  |  |  |  |  |  |  |  |  |  |  |  |
| 10 |  |  |  |  |  |  |  |  |  |  |  |  |  |  |  |  |  |  |  |

**Fig. S2**

| **(A)** | **(B)** |
| --- | --- |
| **(C)** | **(D)** |

**Fig. S3**

| **(A)** | **(B)** |
| --- | --- |
| **(C)** | **(D)** |

**Fig. S4**

| **(A)** | **(B)** |
| --- | --- |
| **(C)** | **(D)** |

**Fig. S5**

| **(A)** | **(B)** |
| --- | --- |
| **(C)** | **(D)** |

**Fig. S6**
